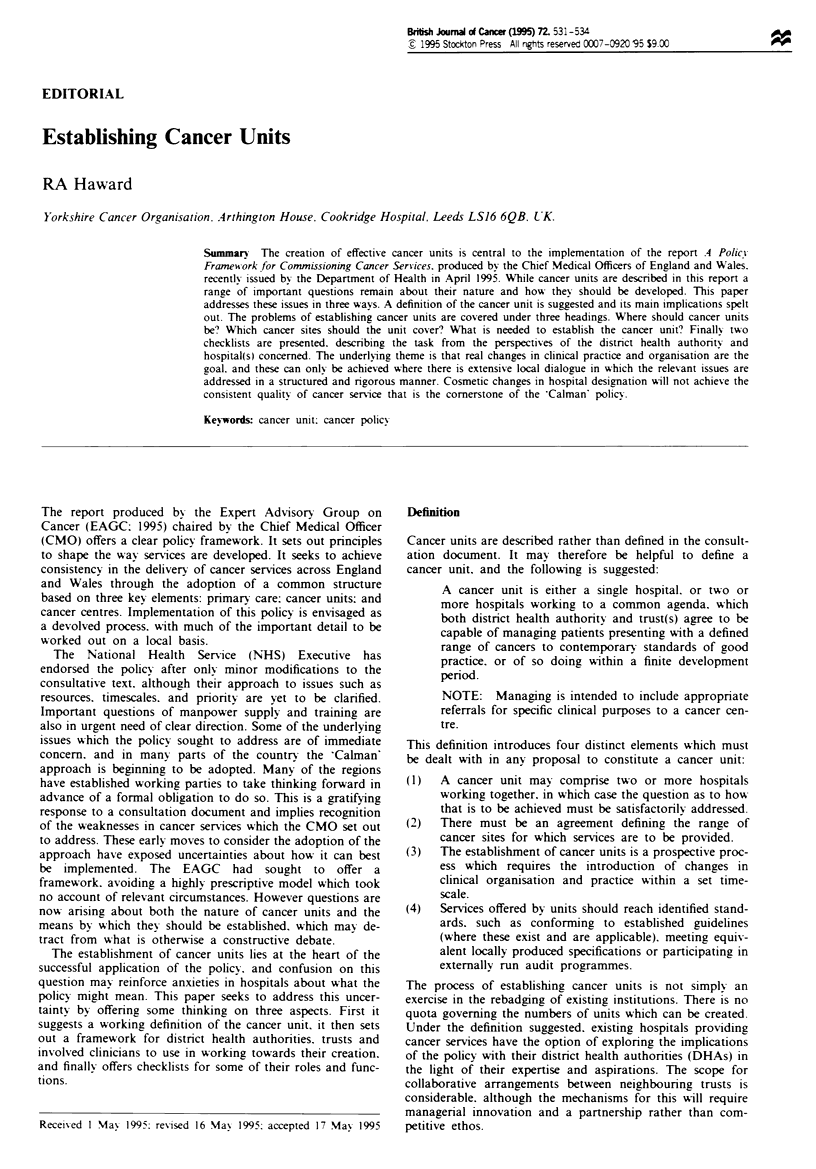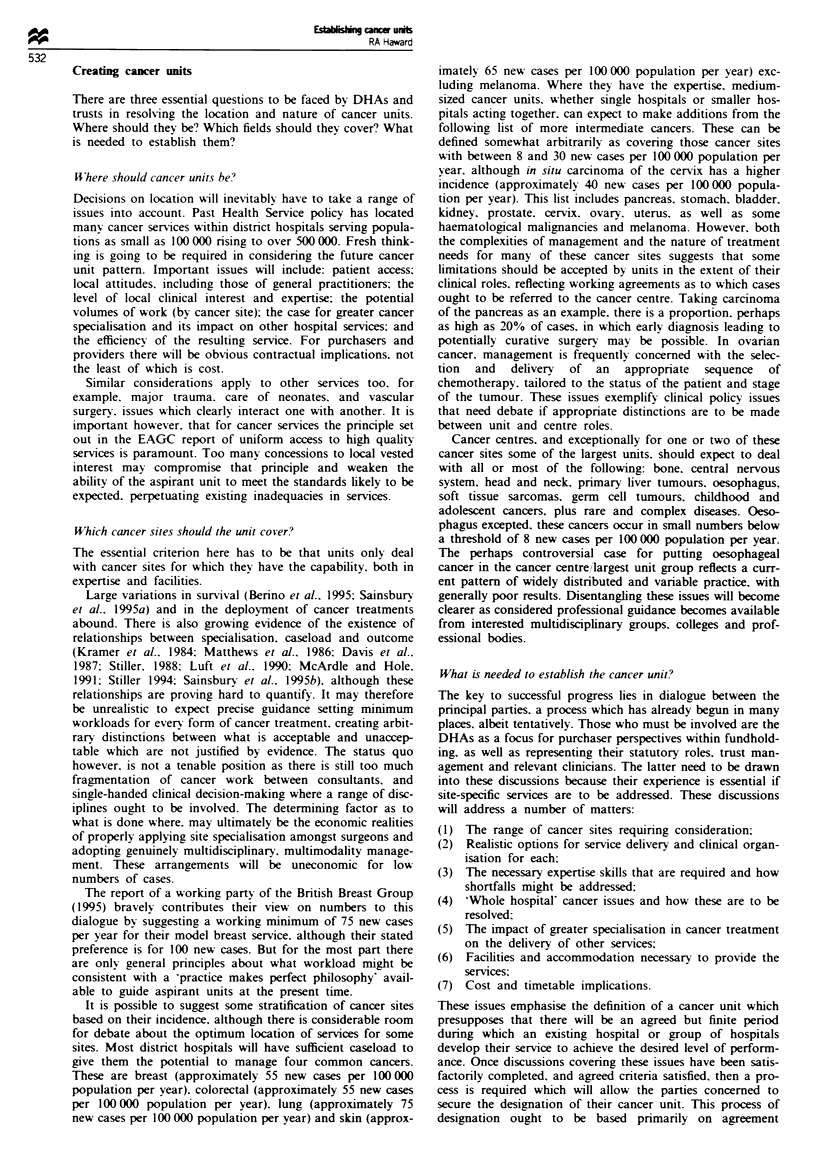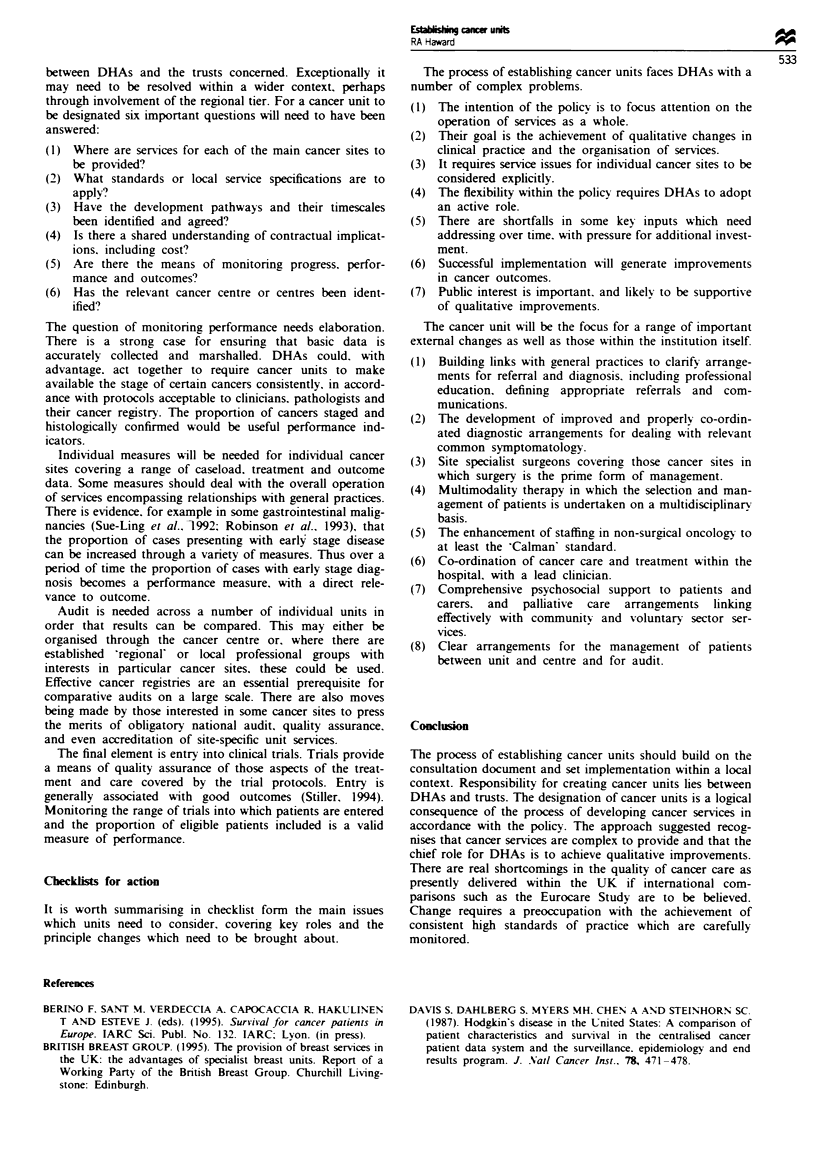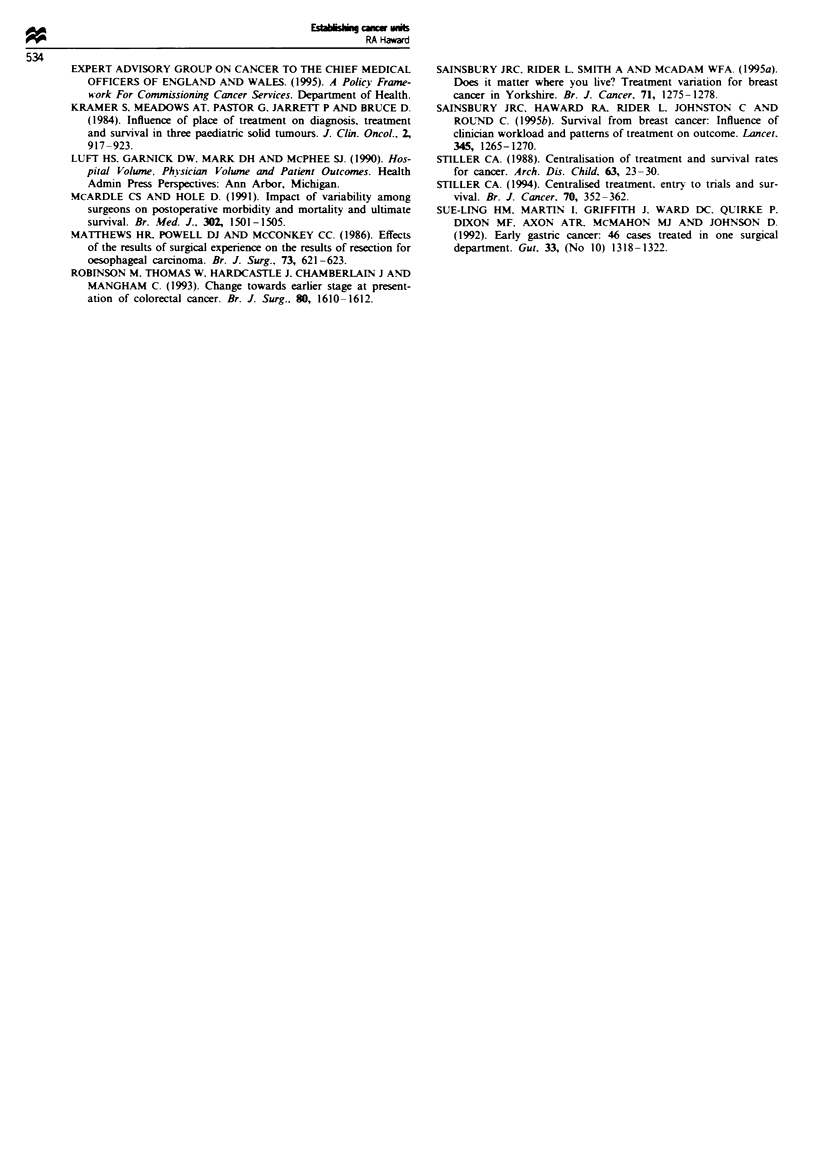# Establishing cancer units.

**DOI:** 10.1038/bjc.1995.369

**Published:** 1995-09

**Authors:** R. A. Haward

## Abstract

The creation of effective cancer units is central to the implementation of the report A Policy Framework for Commissioning Cancer Services, produced by the Chief Medical Officers of England and Wales, recently issued by the Department of Health in April 1995. While cancer units are described in this report a range of important questions remain about their nature and how they should be developed. This paper addresses these issues in three ways. A definition of the cancer unit is suggested and its main implications spelt out. The problems of establishing cancer units are covered under three headings. Where should cancer units be? Which cancer sites should the unit cover? What is needed to establish the cancer unit? Finally two checklists are presented, describing the task from the perspectives of the district health authority and hospital(s) concerned. The underlying theme is that real changes in clinical practice and organisation are the goal, and these can only be achieved where there is extensive local dialogue in which the relevant issues are addressed in a structured and rigorous manner. Cosmetic changes in hospital designation will not achieve the consistent quality of cancer service that is the cornerstone of the 'Calman' policy.


British Journal of Cancer (1995) 72, 531-534

© 1995 Stockton Press All rights reserved 0007-0920/95 $9.00

**EDITORIAL**

### Establishing Cancer Units

RA Haward

*Yorkshire Cancer Organisation, Arthington House, Cookridge Hospital, Leeds LS16 6QB, UK.*

**Summary** The creation of effective cancer units is central to the implementation of the report *A Policy Framework for Commissioning Cancer Services*, produced by the Chief Medical Officers of England and Wales, recently issued by the Department of Health in April 1995. While cancer units are described in this report a range of important questions remain about their nature and how they should be developed. This paper addresses these issues in three ways. A definition of the cancer unit is suggested and its main implications spelt out. The problems of establishing cancer units are covered under three headings. Where should cancer units be? Which cancer sites should the unit cover? What is needed to establish the cancer unit? Finally two checklists are presented, describing the task from the perspectives of the district health authority and hospital(s) concerned. The underlying theme is that real changes in clinical practice and organisation are the goal, and these can only be achieved where there is extensive local dialogue in which the relevant issues are addressed in a structured and rigorous manner. Cosmetic changes in hospital designation will not achieve the consistent quality of cancer service that is the cornerstone of the 'Calman' policy.

**Keywords:** cancer unit; cancer policy

The report produced by the Expert Advisory Group on Cancer (EAGC; 1995) chaired by the Chief Medical Officer (CMO) offers a clear policy framework. It sets out principles to shape the way services are developed. It seeks to achieve consistency in the delivery of cancer services across England and Wales through the adoption of a common structure based on three key elements: primary care; cancer units; and cancer centres. Implementation of this policy is envisaged as a devolved process, with much of the important detail to be worked out on a local basis.

The National Health Service (NHS) Executive has endorsed the policy after only minor modifications to the consultative text, although their approach to issues such as resources, timescales, and priority are yet to be clarified. Important questions of manpower supply and training are also in urgent need of clear direction. Some of the underlying issues which the policy sought to address are of immediate concern, and in many parts of the country the 'Calman' approach is beginning to be adopted. Many of the regions have established working parties to take thinking forward in advance of a formal obligation to do so. This is a gratifying response to a consultation document and implies recognition of the weaknesses in cancer services which the CMO set out to address. These early moves to consider the adoption of the approach have exposed uncertainties about how it can best be implemented. The EAGC had sought to offer a framework, avoiding a highly prescriptive model which took no account of relevant circumstances. However questions are now arising about both the nature of cancer units and the means by which they should be established, which may detract from what is otherwise a constructive debate.

The establishment of cancer units lies at the heart of the successful application of the policy, and confusion on this question may reinforce anxieties in hospitals about what the policy might mean. This paper seeks to address this uncertainty by offering some thinking on three aspects. First it suggests a working definition of the cancer unit, it then sets out a framework for district health authorities, trusts and involved clinicians to use in working towards their creation, and finally offers checklists for some of their roles and functions.

Received 1 May 1995; revised 16 May 1995; accepted 17 May 1995

### Definition

Cancer units are described rather than defined in the consultation document. It may therefore be helpful to define a cancer unit, and the following is suggested:

> A cancer unit is either a single hospital, or two or more hospitals working to a common agenda, which both district health authority and trust(s) agree to be capable of managing patients presenting with a defined range of cancers to contemporary standards of good practice, or of so doing within a finite development period.
>
> NOTE: Managing is intended to include appropriate referrals for specific clinical purposes to a cancer centre.

This definition introduces four distinct elements which must be dealt with in any proposal to constitute a cancer unit:

(1) A cancer unit may comprise two or more hospitals working together, in which case the question as to how that is to be achieved must be satisfactorily addressed.
(2) There must be an agreement defining the range of cancer sites for which services are to be provided.
(3) The establishment of cancer units is a prospective process which requires the introduction of changes in clinical organisation and practice within a set timescale.
(4) Services offered by units should reach identified standards, such as conforming to established guidelines (where these exist and are applicable), meeting equivalent locally produced specifications or participating in externally run audit programmes.

The process of establishing cancer units is not simply an exercise in the rebadging of existing institutions. There is no quota governing the numbers of units which can be created. Under the definition suggested, existing hospitals providing cancer services have the option of exploring the implications of the policy with their district health authorities (DHAs) in the light of their expertise and aspirations. The scope for collaborative arrangements between neighbouring trusts is considerable, although the mechanisms for this will require managerial innovation and a partnership rather than competitive ethos.

### Creating cancer units

There are three essential questions to be faced by DHAs and trusts in resolving the location and nature of cancer units. Where should they be? Which fields should they cover? What is needed to establish them?

### *Where should cancer units be?*

Decisions on location will inevitably have to take a range of issues into account. Past Health Service policy has located many cancer services within district hospitals serving populations as small as 100 000 rising to over 500 000. Fresh thinking is going to be required in considering the future cancer unit pattern. Important issues will include: patient access; local attitudes, including those of general practitioners; the level of local clinical interest and expertise; the potential volumes of work (by cancer site); the case for greater cancer specialisation and its impact on other hospital services; and the efficiency of the resulting service. For purchasers and providers there will be obvious contractual implications, not the least of which is cost.

Similar considerations apply to other services too, for example, major trauma, care of neonates, and vascular surgery, issues which clearly interact one with another. It is important however, that for cancer services the principle set out in the EAGC report of uniform access to high quality services is paramount. Too many concessions to local vested interest may compromise that principle and weaken the ability of the aspirant unit to meet the standards likely to be expected, perpetuating existing inadequacies in services.

### *Which cancer sites should the unit cover?*

The essential criterion here has to be that units only deal with cancer sites for which they have the capability, both in expertise and facilities.

Large variations in survival (Berino *et al*., 1995; Sainsbury *et al*., 1995*a*) and in the deployment of cancer treatments abound. There is also growing evidence of the existence of relationships between specialisation, caseload and outcome (Kramer *et al*., 1984; Matthews *et al*., 1986; Davis *et al*., 1987; Stiller, 1988; Luft *et al*., 1990; McArdle and Hole, 1991; Stiller 1994; Sainsbury *et al*., 1995*b*), although these relationships are proving hard to quantify. It may therefore be unrealistic to expect precise guidance setting minimum workloads for every form of cancer treatment, creating arbitrary distinctions between what is acceptable and unacceptable which are not justified by evidence. The status quo however, is not a tenable position as there is still too much fragmentation of cancer work between consultants, and single-handed clinical decision-making where a range of disciplines ought to be involved. The determining factor as to what is done where, may ultimately be the economic realities of properly applying site specialisation amongst surgeons and adopting genuinely multidisciplinary, multimodality management. These arrangements will be uneconomic for low numbers of cases.

The report of a working party of the British Breast Group (1995) bravely contributes their view on numbers to this dialogue by suggesting a working minimum of 75 new cases per year for their model breast service, although their stated preference is for 100 new cases. But for the most part there are only general principles about what workload might be consistent with a 'practice makes perfect philosophy' available to guide aspirant units at the present time.

It is possible to suggest some stratification of cancer sites based on their incidence, although there is considerable room for debate about the optimum location of services for some sites. Most district hospitals will have sufficient caseload to give them the potential to manage four common cancers. These are breast (approximately 55 new cases per 100 000 population per year), colorectal (approximately 55 new cases per 100 000 population per year), lung (approximately 75 new cases per 100 000 population per year) and skin (approximately 65 new cases per 100 000 population per year) excluding melanoma. Where they have the expertise, medium-sized cancer units, whether single hospitals or smaller hospitals acting together, can expect to make additions from the following list of more intermediate cancers. These can be defined somewhat arbitrarily as covering those cancer sites with between 8 and 30 new cases per 100 000 population per year, although *in situ* carcinoma of the cervix has a higher incidence (approximately 40 new cases per 100 000 population per year). This list includes pancreas, stomach, bladder, kidney, prostate, cervix, ovary, uterus, as well as some haematological malignancies and melanoma. However, both the complexities of management and the nature of treatment needs for many of these cancer sites suggests that some limitations should be accepted by units in the extent of their clinical roles, reflecting working agreements as to which cases ought to be referred to the cancer centre. Taking carcinoma of the pancreas as an example, there is a proportion, perhaps as high as 20% of cases, in which early diagnosis leading to potentially curative surgery may be possible. In ovarian cancer, management is frequently concerned with the selection and delivery of an appropriate sequence of chemotherapy, tailored to the status of the patient and stage of the tumour. These issues exemplify clinical policy issues that need debate if appropriate distinctions are to be made between unit and centre roles.

Cancer centres, and exceptionally for one or two of these cancer sites some of the largest units, should expect to deal with all or most of the following: bone, central nervous system, head and neck, primary liver tumours, oesophagus, soft tissue sarcomas, germ cell tumours, childhood and adolescent cancers, plus rare and complex diseases. Oesophagus excepted, these cancers occur in small numbers below a threshold of 8 new cases per 100 000 population per year. The perhaps controversial case for putting oesophageal cancer in the cancer centre/largest unit group reflects a current pattern of widely distributed and variable practice, with generally poor results. Disentangling these issues will become clearer as considered professional guidance becomes available from interested multidisciplinary groups, colleges and professional bodies.

### *What is needed to establish the cancer unit?*

The key to successful progress lies in dialogue between the principal parties, a process which has already begun in many places, albeit tentatively. Those who must be involved are the DHAs as a focus for purchaser perspectives within fundholding, as well as representing their statutory roles, trust management and relevant clinicians. The latter need to be drawn into these discussions because their experience is essential if site-specific services are to be addressed. These discussions will address a number of matters:

(1) The range of cancer sites requiring consideration;
(2) Realistic options for service delivery and clinical organisation for each;
(3) The necessary expertise/skills that are required and how shortfalls might be addressed;
(4) 'Whole hospital' cancer issues and how these are to be resolved;
(5) The impact of greater specialisation in cancer treatment on the delivery of other services;
(6) Facilities and accommodation necessary to provide the services;
(7) Cost and timetable implications.

These issues emphasise the definition of a cancer unit which presupposes that there will be an agreed but finite period during which an existing hospital or group of hospitals develop their service to achieve the desired level of performance. Once discussions covering these issues have been satisfactorily completed, and agreed criteria satisfied, then a process is required which will allow the parties concerned to secure the designation of their cancer unit. This process of designation ought to be based primarily on agreement between DHAs and the trusts concerned. Exceptionally it may need to be resolved within a wider context, perhaps through involvement of the regional tier. For a cancer unit to be designated six important questions will need to have been answered:

(1) Where are services for each of the main cancer sites to be provided?
(2) What standards or local service specifications are to apply?
(3) Have the development pathways and their timescales been identified and agreed?
(4) Is there a shared understanding of contractual implications, including cost?
(5) Are there the means of monitoring progress, performance and outcomes?
(6) Has the relevant cancer centre or centres been identified?

The question of monitoring performance needs elaboration. There is a strong case for ensuring that basic data is accurately collected and marshalled. DHAs could, with advantage, act together to require cancer units to make available the stage of certain cancers consistently, in accordance with protocols acceptable to clinicians, pathologists and their cancer registry. The proportion of cancers staged and histologically confirmed would be useful performance indicators.

Individual measures will be needed for individual cancer sites covering a range of caseload, treatment and outcome data. Some measures should deal with the overall operation of services encompassing relationships with general practices. There is evidence, for example in some gastrointestinal malignancies (Sue-Ling *et al.*, 1992; Robinson *et al.*, 1993), that the proportion of cases presenting with early stage disease can be increased through a variety of measures. Thus over a period of time the proportion of cases with early stage diagnosis becomes a performance measure, with a direct relevance to outcome.

Audit is needed across a number of individual units in order that results can be compared. This may either be organised through the cancer centre or, where there are established 'regional' or local professional groups with interests in particular cancer sites, these could be used. Effective cancer registries are an essential prerequisite for comparative audits on a large scale. There are also moves being made by those interested in some cancer sites to press the merits of obligatory national audit, quality assurance, and even accreditation of site-specific unit services.

The final element is entry into clinical trials. Trials provide a means of quality assurance of those aspects of the treatment and care covered by the trial protocols. Entry is generally associated with good outcomes (Stiller, 1994). Monitoring the range of trials into which patients are entered and the proportion of eligible patients included is a valid measure of performance.

### Checklists for action

It is worth summarising in checklist form the main issues which units need to consider, covering key roles and the principle changes which need to be brought about.

The process of establishing cancer units faces DHAs with a number of complex problems.

(1) The intention of the policy is to focus attention on the operation of services as a whole.
(2) Their goal is the achievement of qualitative changes in clinical practice and the organisation of services.
(3) It requires service issues for individual cancer sites to be considered explicitly.
(4) The flexibility within the policy requires DHAs to adopt an active role.
(5) There are shortfalls in some key inputs which need addressing over time, with pressure for additional investment.
(6) Successful implementation will generate improvements in cancer outcomes.
(7) Public interest is important, and likely to be supportive of qualitative improvements.

The cancer unit will be the focus for a range of important external changes as well as those within the institution itself.

(1) Building links with general practices to clarify arrangements for referral and diagnosis, including professional education, defining appropriate referrals and communications.
(2) The development of improved and properly co-ordinated diagnostic arrangements for dealing with relevant common symptomatology.
(3) Site specialist surgeons covering those cancer sites in which surgery is the prime form of management.
(4) Multimodality therapy in which the selection and management of patients is undertaken on a multidisciplinary basis.
(5) The enhancement of staffing in non-surgical oncology to at least the 'Calman' standard.
(6) Co-ordination of cancer care and treatment within the hospital, with a lead clinician.
(7) Comprehensive psychosocial support to patients and carers, and palliative care arrangements linking effectively with community and voluntary sector services.
(8) Clear arrangements for the management of patients between unit and centre and for audit.

### Conclusion

The process of establishing cancer units should build on the consultation document and set implementation within a local context. Responsibility for creating cancer units lies between DHAs and trusts. The designation of cancer units is a logical consequence of the process of developing cancer services in accordance with the policy. The approach suggested recognises that cancer services are complex to provide and that the chief role for DHAs is to achieve qualitative improvements. There are real shortcomings in the quality of cancer care as presently delivered within the UK if international comparisons such as the Eurocare Study are to be believed. Change requires a preoccupation with the achievement of consistent high standards of practice which are carefully monitored.

### References

BERINO F, SANT M, VERDECCIA A, CAPOCACCIA R, HAKULINEN T AND ESTEVE J. (eds). (1995). *Survival for cancer patients in Europe*. IARC Sci. Publ. No. 132. IARC: Lyon. (in press).

BRITISH BREAST GROUP. (1995). The provision of breast services in the UK: the advantages of specialist breast units. Report of a Working Party of the British Breast Group. Churchill Livingstone: Edinburgh.

DAVIS S, DAHLBERG S, MYERS MH, CHEN A AND STEINHORN SC. (1987). Hodgkin's disease in the United States: A comparison of patient characteristics and survival in the centralised cancer patient data system and the surveillance, epidemiology and end results program. *J. Natl Cancer Inst.*, **78**, 471–478.

EXPERT ADVISORY GROUP ON CANCER TO THE CHIEF MEDICAL OFFICERS OF ENGLAND AND WALES. (1995). *A Policy Framework For Commissioning Cancer Services*. Department of Health.

KRAMER S, MEADOWS AT, PASTOR G, JARRETT P AND BRUCE D. (1984). Influence of place of treatment on diagnosis, treatment and survival in three paediatric solid tumours. *J. Clin. Oncol.*, **2**, 917-923.

LUFT HS, GARNICK DW, MARK DH AND MCPHEE SJ. (1990). *Hospital Volume, Physician Volume and Patient Outcomes*. Health Admin Press Perspectives: Ann Arbor, Michigan.

MCARDLE CS AND HOLE D. (1991). Impact of variability among surgeons on postoperative morbidity and mortality and ultimate survival. *Br. Med. J.*, **302**, 1501-1505.

MATTHEWS HR, POWELL DJ AND MCCONKEY CC. (1986). Effects of the results of surgical experience on the results of resection for oesophageal carcinoma. *Br. J. Surg.*, **73**, 621-623.

ROBINSON M, THOMAS W, HARDCASTLE J, CHAMBERLAIN J AND MANGHAM C. (1993). Change towards earlier stage at presentation of colorectal cancer. *Br. J. Surg.*, **80**, 1610-1612.

SAINSBURY JRC, RIDER L, SMITH A AND MCADAM WFA. (1995*a*). Does it matter where you live? Treatment variation for breast cancer in Yorkshire. *Br. J. Cancer*, **71**, 1275-1278.

SAINSBURY JRC, HAWARD RA, RIDER L, JOHNSTON C AND ROUND C. (1995*b*). Survival from breast cancer: Influence of clinician workload and patterns of treatment on outcome. *Lancet*, **345**, 1265-1270.

STILLER CA. (1988). Centralisation of treatment and survival rates for cancer. *Arch. Dis. Child*, **63**, 23-30.

STILLER CA. (1994). Centralised treatment, entry to trials and survival. *Br. J. Cancer*, **70**, 352-362.

SUE-LING HM, MARTIN I, GRIFFITH J, WARD DC, QUIRKE P, DIXON MF, AXON ATR, MCMAHON MJ AND JOHNSON D. (1992). Early gastric cancer: 46 cases treated in one surgical department. *Gut*, **33**, (No 10) 1318-1322.